# Neuroserpin, IL-33 and IL-17A as potential markers of mild symptoms of depressive syndrome in *Toxoplasma gondii*-infected pregnant women

**DOI:** 10.3389/fimmu.2024.1394456

**Published:** 2024-05-17

**Authors:** Zolder Marinho Silva, Débora Nonato Miranda Toledo, Sirlaine Pio, Bianca Alves Almeida Machado, Priscilla Vilela dos Santos, Flávia Galvão Hó, Yasmim Nogueira Medina, Paulo Henrique de Miranda Cordeiro, Luiza Oliveira Perucci, Kelerson Mauro de Castro Pinto, André Talvani

**Affiliations:** ^1^ Laboratório de Imunobiologia da Inflamação, Departamento de Ciências Biológicas/ICEB, Universidade Federal de Ouro Preto, Ouro Preto, MG, Brazil; ^2^ Programa de Pós-Graduação em Saúde e Nutrição, Universidade Federal de Ouro Preto, Ouro Preto, MG, Brazil; ^3^ Programa de Pós-Graduação em Evolução Crustal e Recursos Naturais, Universidade Federal de Ouro Preto, Ouro Preto, MG, Brazil; ^4^ Escola de Medicina, Universidade Federal de Ouro Preto, Ouro Preto, MG, Brazil; ^5^ Department of Obstetrics Gynecology and Reproductive Sciences, California University, San Diego, CA, United States; ^6^ Escola de Educação Física, Universidade Federal de Ouro Preto, Ouro Preto, MG, Brazil; ^7^ Programa de Pós-Graduação em Infectologia e Medicina Tropical, Universidade Federal de Minas Gerais, Belo Horizonte, MG, Brazil

**Keywords:** pregnant women, depressive syndrome, neuroserpin, IL-33, toxoplasma gondii

## Abstract

**Introduction:**

Depressive syndrome (DS) is a common complication during pregnancy and the postpartum period, and is triggered by multiple organic/genetic and environmental factors. Clinical and biochemical follow-up is essential for the early diagnosis and prognosis of DS. The protozoan Toxoplasma gondii causes infectious damage to the fetus during parasite primary-infection. However, in long-term infections, pregnant women develop immune protection to protect the fetus, although they remain susceptible to pathological or inflammatory effects induced by T. gondii. This study aimed to investigate plasma inflammatory biomarkers in pregnant women seropositive and seronegative for T. gondii, with diagnoses of minor and moderate/severe DS.

**Methods:**

Pregnant women (n=45; age=18–39 years) were recruited during prenatal care at health centers in Ouro Preto, Minas Gerais, Brazil. Participants were asked to complete a socio-demographic questionnaire to be submitted to well-standardized DS scale calculators (Beck Depression Inventory Questionnaire, Edinburgh Postnatal Depression Scale, and Major Depressive Episode Module). Additionally, 4 mL of blood was collected for plasma neuroserpin, CCL2, IL-17A, and IL-33 analysis.

**Results:**

Pregnant volunteers with chronic T. gondii contact were all IgG+ (44%; n=21) and exhibited increased plasma IL-33, IL-17A, and neuroserpin levels, but not CCL2, compared to uninfected pregnant women. Using Beck’s depression inventory, we observed an increase in plasma IL-17A and IL-33 in women with T. gondii infeCction diagnosed with mild DS, whereas neuroserpin was associated with minor and moderate/severe DS.

**Discussion:**

Our data suggest a close relationship between DS in pregnant women with chronic T. gondii infection and neurological conditions, which may be partially mediated by plasma neuroserpin, IL-33, and IL-17A levels.

## 1 Introduction

Toxoplasmosis, a widely disseminated zoonosis, is caused by the intracellular parasite *Toxoplasma gondii*, which is found in warm-blooded animals, including humans. Transmission occurs through the ingestion of oocysts shed by infected felines or the ingestion of cysts (bradyzoites) in fresh meats (1, 2). In immunocompromised individuals, primo-infected pregnant women, and immunocompetent individuals, there are significant risks to the host’s life, including death, malformation, abortion, and ocular and neurological disturbances (3–5).

*T. gondii* sustains a lifelong presence in the central nervous system, altering neurological structures and neurotransmitters, and inducing behavioral and cognitive changes that facilitate the predation of infected hosts, thereby maintaining the parasite’s life cycle (6). Furthermore, during the chronic stage of infection, the immune response within the central nervous system may potentiate neuronal plasticity and other cognitive patterns in infected hosts (7). Alternatively, it is suggested that *T. gondii* manipulates host behavior to increase the transmission rates by infected brain cells (8). Latent toxoplasmosis is also associated with the development of schizophrenia, anxiety disorder, aggressivity and impulsivity, suicidal attempts and depression (9). Depression, in particular, is suggested to be related to IFN-γ blocking during *T. gondii* growth, which occurs by inducing indoleamine-2,3-dioxygenase activation and tryptophan depletion, causing a reduction of serotonin in the central nervous system area (10).

Depressive syndrome (DS) is a mood mental/cognitive disorder characterized by persistent feelings of sadness and loss of pleasure or interest in activities, affecting thousands of individuals worldwide (11, 12). Physiological conditions that alter hormone and neurotransmitter networks, such as premenstrual and menopause syndromes and pregnancy, can trigger or exacerbate DS (13, 14). Pregnancy, typically a 40-week period during which the fetus develops inside a woman’s womb or uterus, is accompanied by significant hormonal and physiological alterations in the pregnant woman (15, 16). DS occurs in 1–5 pregnancies, with a higher frequency during the prenatal period. Symptoms include a loss of humor and motivation, anxiety, feelings of guilt, sadness, and suicidal thoughts (17).

Chronic *T. gondii* infection may trigger neurological disturbances, and the host immune response to parasites can contribute to central nervous system disorders. Therefore, this study aimed to evaluate neuroserpin, IL-33, IL-17A, and CCL2 as biomarkers for DS in Brazilian pregnant women with *T. gondii* infection.

## 2 Materials and methods

### 2.1 Study population

A cross-sectional study was conducted between December 2020 and October 2021. A total of 45 pregnant women volunteered, and their plasma levels of specific immunoglobulin (Ig)M and IgG antibodies against *T. gondii* were evaluated at the Pilot Laboratory of the Pharmacy School at the Federal University of Ouro Preto (UFOP). A microparticle enzyme Immunoassay was used to detect anti-*T. gondii* IgG and IgM antibodies in biological samples. Reference values for IgG were: > 3 UI/mL: reactive; between 2-3 UI/mL: indeterminate; and < 2 UI/mL: nonreactive. Reference values for IgM were: > 0.600 UI/mL: reactive; between 0.500-0.600 UI/mL: indeterminate; and < 0.500 UI/mL: nonreactive (18). Sociodemographic, environmental, and gestational patterns were investigated using a semi-structured questionnaire (Santos et al., 2023) after clinical attendance.

To ascertain the incidence of DS in pregnant women, the volunteers completed a formally structured questionnaire/interview based on the Diagnostic and Statistical Manual of Mental Disorders (DSM-IV and TR) (19), and included the Beck Depression Inventory Questionnaire, Edinburgh Postnatal Depression Scale, and Major Depressive Episode Module.

This study was approved by the Institutional Research Ethics Committee (UFOP CAAE:23467219.7.0000.5150). All volunteers provided a signed the Informed Consent Term. For pregnant participants under 18 years of age, a parent or guardian also provided consent by signing the form.

### 2.2 Immunological analysis

Venous blood samples (4 mL) were collected in polypropylene tubes - Vacuette (GreinerBio-One, Kremsmünster, Austria) containing EDTA. The samples were centrifuged (3500 ×*g*, 4°C, 10 min) to separate the plasma, which was then aliquoted and stored at −80°C in an ultra-freezer until the immunoassays (ELISA) were performed.

According to the manufacturer’s protocols, plasma biomarkers were measured, in duplicate, using quantitative human ELISA kits for IL-17A, IL-33, CCL2, and neuroserpin from PeproTech ^®^ (New Jersey, USA). The absorbance reading was performed on a microplate reader (SpectraMax^®^ 190, Molecular Devices, CA, USA), using 450/630nm ratio of wavelength.

### 2.3 Statistical analysis

GraphPad Prism 8 (GraphPad, San Diego, CA, EUA) was used for data analysis in this study. To evaluate differences in plasma biomarkers and the DS questionnaires, the Shapiro-Wilk normality test was performed. For normally distributed data, the Student’s t-test was performed and the Mann-Whitney test was performed for non-parametric data. Statistical significance was set at p < 0.05.

## 3 Results

Among 45 pregnant women, 24 were seronegative (IgM- and IgG-) for *T. gondii*, whereas 21 were chronically seropositive (IgG+). In the seropositive women, IgM levels were low (index < 0.5 UI/mL), whereas levels of IgG were > 3,0 UI/mL, indicating the chronic nature of the infection in these patients.

According to the sociodemographic questionnaire, the majority of the pregnant women were single (71.1%), 57.8% had multiple children, and 17.8% reported experiencing spontaneous abortion. The questionnaire also revealed that 46.7% of the pregnant women were overweight or obese, 48.9% were in their second gestational trimester, and 33.3% owned cats with free-roaming habits. Additionally, 95.6% reported consuming uncooked vegetables, and 37.8% washed food under running water (**Table 1**).

**Table 1:** Univariate analysis with the characterization of pregnant women evaluated in 2020/2021- Ouro Preto, MG, Brasil.

Variables	N	%	*T. gondii*		
			Uninfected pregnant women (n=24)	Infected pregnant women (n=21)	*p-value*
Socioeconomic variables					
Age					
Adolescents (<18 years)	3	6,6	2	1	>0,999^a^
Adults (>18 years)	42	93,4	22	20	
Education					
< Complete high school	15	33,3	7	8	0,5463^a^
> Complete high school	30	66,7	17	13	
Income					
Unkown	4	8,9	3	1	0,5541^c^
Up to 1 minimum wage	21	46,7	12	9	
1-3 minimum wage	13	28,9	5	8	
3-5 minimum wage	6	13,3	3	3	
5-15 minimum wage	1	2,2	1	0	
Civil status					
Single	32	71,1	16	16	0,5284^a^
Married/ Former civil partner	13	28,9	8	5	
Environmental variables					
Access to sewer services					
No	2	4,4	0	2	0,2121^a^
Yes	43	95,6	24	19	
Pets in the house					
No	19	42,2	10	9	0,1250^d^
Yes	26	57,8	14	12	
Contact with pets					
No	28	62,2	18	10	0,0727^a^
Yes	17	37,8	6	11	
Animal waste disposed of in the house					
No	32	71,1	17	15	>0,9999^a^
Yes	13	28,9	7	6	
Oocyst transmission vehicle					
No	30	66,7	14	16	0,3421^a^
Yes	15	33,3	10	5	
Dietary variables					
Unpasteurized milk consumption					
No	42	93,3	23	19	0,5915^a^
Yes	3	6,7	1	2	
Raw vegetables consumption					
No	2	4,4	2	0	0,4909^a^
Yes	43	95,6	22	21	
Raw meat consumption					
No	35	77,8	20	15	0,4764^a^
Yes	10	22,2	4	6	
Fruit and vegetable washing process					
Does not sanitize	5	11,1	3	2	0,4028^c^
Sanitizes under running water	17	37,8	7	10	
Sanitizes with hypochlorite	14	31,1	8	6	
Others	9	20,0	6	3	
Gestational variables / Mental heath					
Gestational age					
First trimester	8	17,8	5	3	
Second trimester	22	48,9	11	11	0,8318^b^
Third trimester	15	33,3	8	7	
Pregnancy					
Primigravida	19	42,2	12	7	0,3661^a^
Multigravida	26	57,8	12	14	
Abortion					
No	37	82,2	21	16	0,4427^a^
Yes	8	17,8	3	5	
Pre-gestational BMI					
Not Overweight	19	42,2	12	7	
Overwight	22	48,9	10	12	0,3447^c^
Not informed	4	8,9	2	2	
Gestational weight gain					
Ideal	1	2,2	1	0	
Low	21	46,7	12	9	
Excessive	18	40,0	8	10	0,5567^c^
Not informed	5	11,1	3	2	
Health problems / Comorbidities					
No	34	75,6	19	15	0,7302^a^
Yes	11	24,4	5	6	
Medication use					
No	38	84,4	20	18	>0,9999^a^
Yes	7	15,6	4	3	
Family history (mental health of family members)					
No	32	71,1	17	15	>0,9999^a^
Yes	13	28,9	7	6	
Past medical history (Depression history)					
No	30	66,7	17	13	0,5463^a^
Yes	15	33,3	7	8	
Postpartum depression in previous pregnancy					
No	23	88,4	11	12	>0,9999^a^
Yes	3	11,6	1	2	

a Fisher's exact test.

b Pearson's chi-square test.

c Chi-square partition test.

d Wilconox test.

Based on the questionnaire/interview focusing on DS, 27.7% of the volunteers reported a family history of mental health issues, 36.1% disclosed previous episodes of depression, and 8.5% had experienced postpartum depression (**Table 2**). Regarding DS, 22.2% of the volunteers exhibited moderate to severe symptoms, whereas 28.9% experienced DS exclusively after childbirth (as measured by the Edinburgh scale). Additionally, 31.1% presented with increased and recurrent episodes of depression (as indicated by the EDM scale).

**Table 2:** Mental health analysis of pregnant women evaluated in 2020/2021- Ouro Preto, MG, Brasil.

Questionnaires	N	%	Pregnant women (n=45)		*p-value*
			Uninfected (n=24)	*T. gondii* (n=21)	
Beck Depression Inventory (BDI-II)					
Minimal/mild depression	35	77,8	18	17	0,7289
Moderate/severe depression	10	22,2	6	4	
Edinburgh Depression Research Self-Assessment					
Negative PPD	32	71,1	17	15	>0,9999
Positive PPD	13	28,9	7	6	
Current and Recurrent Major Depressive Episode (MDE)					
Negative MDE	31	68,9	18	13	0,5197
Positive MDE	14	31,1	6	8	

Postpartum depression (PPD) Major depressive episode (MDE) Fisher's exact test.

**Figure 1:**
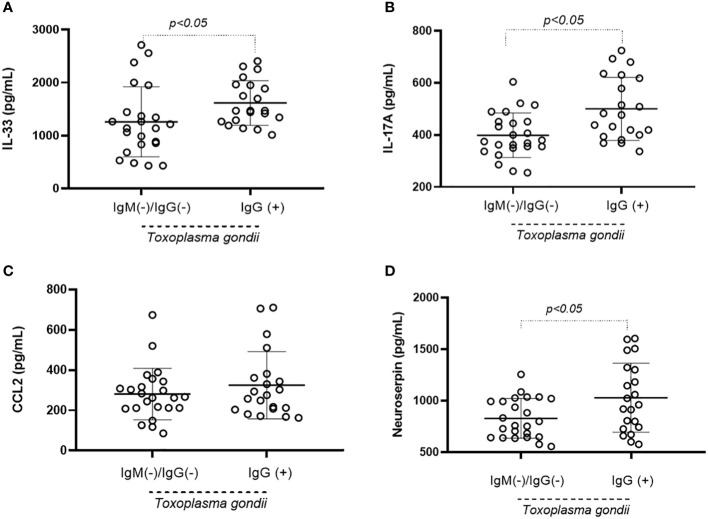
Plasma inflammatory mediators in pregnant women based on *T. gondii* serotyping. Pregnant women were categorized as seronegative (IgM- and IgG-) and as seropositive (IgG+) for *T. gondii* and plasma concentrations of IL-33 **(A)**, IL-17A **(B)**, CCL2 **(C)** and neuroserpin **(D)** presented. Statistical analysis was performed using the Mann-Whitney test. Mean, mean of concentrations; S. E, Std. mean error.

**Figure 2:**
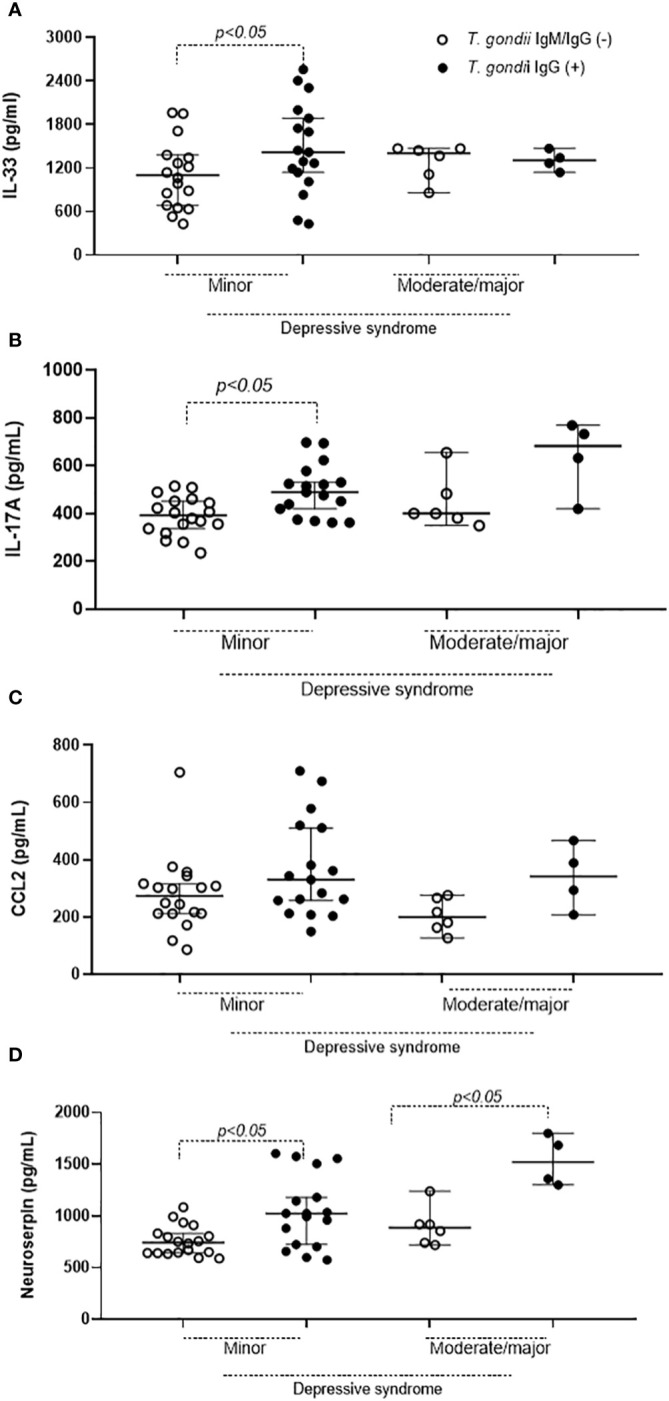
Plasma inflammatory mediators in pregnant women according to serotyping for *T. gondii* and classification by Beck Depression Inventory (DBI-II). The pregnant women were diagnosed as seronegative (IgM- and IgG-) and as seropositive (IgG+) for *T. gondii* and concentrations of IL-33 **(A)**, IL-17A **(B)**, CCL2 **(C)** and neuroserpin **(D)** measured in plasma samples. Statistical analysis was performed using the Student’s T test for parametric data and the Mann-Whitney test for non-parametric data. Mean, mean of concentrations; S.E, Std. mean error.

Upon evaluating plasma biological markers, IL-33 (**Figure 1A**), IL-17A (**Figure 1B**), and neuroserpin (**Figure 1D**) were found to be elevated in the chronic presence of *T. gondii*. This pattern was not observed for the chemokine CCL2 (**Figure 1C**). Moreover, the data on these inflammatory mediators were re-distributed based on the results from the Beck Depression Inventory Questionnaire, the Edinburgh Postnatal Depression Scale, and the Major Depressive Episode Module. The Beck Depression Inventory Questionnaire data revealed that plasma concentrations of IL-33 (**Figure 2A**) and IL-17A (**Figure 2B**) were higher in *T. gondii*-infected pregnant women diagnosed with mild DS. Neuroserpin was the sole inflammatory marker with increased plasma levels in infected women diagnosed with both mild and moderate or severe DS (**Figure 2D**), whereas no difference was observed in CCL2 levels between the two groups (**Figure 2C**). Data obtained from the Edinburgh Postnatal Depression Scale and the Major Depressive Episode Module did not reveal any differences in plasma inflammatory mediators (data not shown).

## 4 Discussion

DS has emerged as a pandemic disorder that affects individuals across different ages, genders, and socioeconomic and cultural backgrounds in recent decades, reaching its peak following the COVID-19 pandemic (20). Genetic risk factors for DS may accelerate the onset of the disorder through the activation of endocrine and/or environmental stimuli or stress (21–23). There is compelling evidence that the immune response plays a significant role in the neurobiological underpinnings of depression, altering the anatomy and function of neurons and modifying neurotransmitters and neuronal synaptic plasticity (24–26).

Beyond the genetic factors contributing to DS, a pathogenic hypothesis has garnered new support which characterizes DS as a host-parasite nonadaptive condition, supported by genes (27). DS manifests as an inflammatory condition mediated by pathogen-associated molecular patterns. These patterns activate innate immune mediators, including the nuclear factor Kappa B and extracellular-signal-regulated kinases pathways, within the central nervous system (28). In this context, *T. gondii* has been highlighted as a protozoan that preferentially infects nucleated cells of the central nervous system, both *in vitro* and in immunodeficient and immunocompetent individuals (29). It is estimated that > 60% of the global population has a chronic central nervous system *T. gondii* infection, which affects children, men, and women, including those in the gestational period (30).

The gestational cycle is a critical phase in women´s lives, as they experience physical, hormonal, psychic, and social changes that generally have a direct effect on their mental health. A limitation of this study was the no dosage of Vitamins B1, B3, B6, B9 and B12 in these pregnant women, since they are essential for neuronal function and for depressive syndrome symptoms. The precocious supplementation of Vitamin B12 has been shown to delay the onset of depressive symptoms and, improve the effects of anti-depressive therapies (31, 32). However, no one volunteer subject was diagnosed with anaemia in this present study, according to the hemogram. Despite this being the case after the first pregnancy, 10–15% of women experience mild, moderate, or severe anxiety and depressive symptoms, including feelings of guilt and a lack of appetite and energy to move forward (33). In pregnant women with DS, high plasma levels of inflammatory mediators, such as IL-1b, IL-1, and IL-6, have been observed (34, 35). A similar immunological pattern has been observed in non-pregnant individuals diagnosed with severe DS, where IL-1b, IL-6, Tumor Necrosis Factor, and C-reactive protein levels are elevated. Interestingly, after partially blocking these inflammatory mediators, there is a documented reduction in depressive symptoms.

In this study, DS was investigated in pregnant women with chronic *T. gondii* infection. We observed elevated levels of three mediators–neuroserpin, IL-33, and IL-17A–in women with mild DS. Previously, our group demonstrated the involvement of IL-33, CCL2, and IL-17A in pregnant women with *T. gondii* infection (36–38). However, neuroserpin was a novel target of this investigation in the context of *T. gondii* infection and was the only marker elevated in plasma of pregnant women with *T. gondii* infection having both mild and moderate/severe DS.

Neuroserpin is a serine protease inhibitor associated with synapse formation and neurogenesis, cellular adhesion and vascular permeability in the central nervous system, and inhibition of the tissue plasminogen activator, and acts as a protective factor against neurological disturbances (39–42). In the context of gestation, neuroserpin has been proposed to be an important mediator in early-onset severe preeclampsia (43). Regarding DS, one study associated neuroserpin with the fibrinolytic system, which performs essential neurological functions. Rats exposed to chronic and unpredictable mild stress, as well as individuals with mild depression, exhibited reductions in neuroserpin mRNA and tissue plasminogen activator in tissues and peripheral blood mononuclear cells (44). In our study involving pregnant women, *T. gondii* infection bias increased neuroserpin concentration in the presence of mild to severe DS, suggesting a potential neuroprotective role of neuroserpin due to a long-term host-parasite relationship.

The alarmin IL-33, IL-17A, and the chemokine CCL2 have been highlighted in studies of depressive disorders (45–47), and their high production/expression was demonstrated in chronic *T. gondii* research in both experimental models and humans (38, 48–50). IL-33, which shows high expression in glial cells and astrocytes, has been associated with neurological diseases such as Alzheimer’s, post-traumatic stress disorder, major depressive disorder, and schizophrenia (51, 52). IL-17A can induce depression-like symptoms through the NF-kB and p38MAPK pathways in mice (53) and hospitalized patients (54). Both IL-33 and IL-17A have been identified as potential prognostic markers in human toxoplasmosis and depressive disorders, as evidenced by their high plasma levels in pregnant women with *T. gondii* infection having mild DS.

CCL2 is a potential chemokine for monocytes and various immune cells via its CCR2 receptor. Generally, CCL2 shows a local and systemic activity associated with *T. gondii* elimination in experimental models and humans, and its elevated expression and production correspond with the chronic state of toxoplasmosis of the central nervous system (49, 55). CCL2 upregulation is also implicated in depressive disorders (56, 57); however, its plasma concentration in pregnant women with *T. gondii* infection having DS did not reach statistical significance in this study.

The IL-17A (58), IL-33 (59), CCL2 (60) and Neuroserpin (42, 43) are maternal inflammatory markers released to the improvement of pregnancy outcomes, neurological and immunological development in fetuses in normal conditions, in the experimental models and human subjects. After recognition of *T. gondii* by the maternal immune system and, activation and recruitment of a new repertoire of inflammatory cells, a new inflammatory environment must be established by the release of a new set of inflammatory and regulatory mediators (including IL-17A, IL-33, CCL2 and neuroserpin), which are essential to contain parasites and, to mitigate the fetus infection ameliorating the prognosis of *T. gondii*-infected newborns (37).

Finally, sociocultural status, lifestyle, and environmental factors, including food quality and hygiene, play a crucial role in the physiological and mental health of pregnant women globally. In particular, toxoplasmosis and immune system patterns can alter physiological and mental health in affected individuals (9, 10, 48). Physical and emotional factors can contribute to perinatal depression, independent of *T. gondii* infectious status, such as demands at work, familiar traumatic experiences and fear or insecurity about childbirth and the care of the new baby. In addition, reduced neuroplasticity and neurocircuitry activity changes in prefrontal dorsolateral cortex with hypofunction of the left dorsolateral prefrontal cortex, together with dysfunctional fronto-limbic control mechanisms exert an important role in the onset and development of depression (61). However, *T. gondii* can infect any area of the brain related to depressive syndrome (frontal lobe, thalamus, hippocampus, striatum, temporal lobe and amygdala), and, in practice, there is no how to predict where this parasite cist has been forming until the clinical manifestations. The presence of *T. gondii* cist might coordinate a multi-variable local immune response that changes the functionality and behaviour of neurological cells from multiple brain regions involved in DS (62). In advance, the regulation of a specific biomarker or its precocious association with DS in T*. gondii* could improve clinical management with pregnant women. In summary, further studies are necessary to confirm whether chronic human toxoplasmosis might exacerbate DS in pregnant women and, to evaluate the potentiality of neuroserpin, IL-33, and IL-17 as biomarkers for DS in *T. gondii* infection focusing on pregnant women from different genetic backgrounds.

## Data availability statement

The raw data supporting the conclusions of this article will be made available by the authors, without undue reservation.

## Ethics statement

The studies involving humans were approved by Institutional Research Ethics Committee (Federal University of Ouro Preto) Register number: CAAE:23467219.7.0000.5150. The studies were conducted in accordance with the local legislation and institutional requirements. Written informed consent for participation in this study was provided by the participants’ legal guardians/next of kin.

## Author contributions

ZS: Conceptualization, Investigation, Methodology, Resources, Writing – original draft. DD: Conceptualization, Data curation, Formal analysis, Investigation, Methodology, Resources, Writing – original draft. SP: Investigation, Methodology, Resources, Writing – review & editing. BM: Investigation, Project administration, Resources, Writing – review & editing. PS: Investigation, Methodology, Resources, Writing – review & editing. FH: Investigation, Methodology, Writing – review & editing. YM: Investigation, Methodology, Resources, Writing – review & editing. PC: Data curation, Methodology, Writing – review & editing. LP: Data curation, Formal analysis, Investigation, Writing – review & editing. KP: Data curation, Methodology, Visualization, Writing – review & editing. AT: Conceptualization, Data curation, Formal analysis, Funding acquisition, Project administration, Supervision, Writing – original draft, Writing – review & editing.
